# The influence of children’s emotional comprehension on peer conflict resolution strategies

**DOI:** 10.3389/fpsyg.2023.1142373

**Published:** 2023-03-14

**Authors:** Yanfei Cao, Haowei Wang, Yaojian Lv, Dongjie Xie

**Affiliations:** ^1^College of Children’s Development and Education, Zhejiang Normal University, Hangzhou, China; ^2^Dongyang Experimental Kindergarten, Dongyang, China

**Keywords:** influence, children, emotional comprehension, peer conflict, resolution strategies

## Abstract

Peer conflicts commonly happen in children’s daily interaction, and strategies they employed to deal with the conflicts have an impact on peer conflict resolution. It has been illustrated that children’s understanding of emotion plays an important role in social communication. However, there is little research focusing on the relation between emotional comprehension and peer conflict resolution strategies. In this study, 90 children of 3 to 6 finished the Test of Emotional Comprehension, and their preschool teachers were required to complete the Conflict Resolution Strategy Questionnaire, which scored each child’s conflict resolution strategies. The results showed that: (a) the preference of conflict resolution strategies differed in age, and girls tended to adopt positive strategies; (b) children’s emotional comprehension grew with age; and (c) children’s conflict resolution strategies and emotional comprehension were closely related. Children’s emotional comprehension can positively predict the overall conflict resolution strategies and negatively predicted negative strategies, whereas mental emotional comprehension can positively predict positive strategies. The factors affecting children’s emotional comprehension and conflict resolution strategies, and their relationship were discussed in depth.

## 1. Introduction

Peer conflict, refers to mutual opposition between people of equal power, and occurs with the increase of children’s peer interaction (Noakes and Rinaldi, 2006; Sidorowicz and Hair, 2009; Liao et al., 2014). The previous studies have examined peer conflict issues from multiple perspectives, mostly focusing on the definition and structure of children’s conflict, including reasons, styles, resolution strategies, characteristics, significance, and the influential factors (Sheehan and Wheeler, 2012; Raikes et al., 2013). Researchers have documented that individual differences in peer sociometric status and teachers’ reactions to disruptive behavior resulted in peer conflict; also, it was considered an essential developmental task as children mature (Miller and Olson, 2000). If the conflict is not properly resolved, it is likely to damage the relationship between children, hinder the cultivation of children’s prosocial behavior, and even affect children’s physical and mental health. Conflict resolution strategies were strategies used for managing conflicts. Since the original conflict model was proposed by Blake and Mouton, researchers provided several instruments to measure conflict strategies, e.g., Rahim developed the Rahim Organizational Conflict Inventory-II (ROCI-II) to measure interpersonal conflict resolution strategies--integrating, obliging, dominating, avoiding, and compromising (Rahim, 1983; Rahim and Magner, 1995; Cai, 2015). Compared with adults, children’s ability of conflict resolving is developing, and is usually associated with children’s understanding of social conventions, cognitive abilities, mastery of expressive language and background of growth (Killen and Turiel, 1991). Family backgrounds are associated with children’s perceptions of peer conflict resolution strategies, e.g., children’s disposition to use negative conflict strategies and aggressive behaviors against peers is likely to be learned from parents (Schudlich et al., 2004). Children who experience positive forms of conflict resolution in family prefer the same strategies with interaction outside the family. The more tolerant the parents are, the more children will take peaceful settlement, exchange and other positive strategies; The more parents punish children, the more children will use force, aggression and other negative strategies. Children are likely to adopt similar strategies to their parents when solving peer conflicts (Lee et al., 2022). Rubin et al. (1998) conducted a follow-up study of children aged 2 to 4, and concluded that negative parenting might lead to a greater tendency to adopt overtly aggressive or destructive solutions. Simultaneously, children’s resolution strategies are situated in conflict process. The perception, understanding and identification of the situation affect the responsive behaviors. Thornberg (2006) found the preschool children’s resolution strategies were influenced by the opponent’s aggressive or non-aggressive responses towards them. Thus, resolution skill learning and intervention from other peers and adults are necessary to facilitate children’s peer conflict resolution, such as teacher training, and drama programs on peer-to-peer conflict for children (Catterall, 2007; Doppler-Bourassa et al., 2008; Majorano et al., 2015). As children mature, their experience in interacting with peers increases, and their social skills get improved (Chen et al., 2001; Sheehan and Wheeler, 2012). Then, they are able to display better resolution strategies and are more inclined to use strategies such as cooperation and negotiation (Ohbuchi and Yamamoto, 1990; Laursen et al., 2001).

Emotional comprehension is a component of emotional competence, which refers to understanding of perspective taking, desire believes, intentions related to emotions of themselves and other’s (Lagattuta and Hansen, 2014; Rocha et al., 2015). Arterberry et al. (2020) insisted that emotional comprehension consists of understanding of the external causes, emotional states and expectations of emotions. Additionally, there may be a relationship between emotional comprehension and social ability. Children aged 3 to 5 developed their emotional comprehension ability faster, and 4-year-old is the critical developmental stage of emotional comprehension (Chen et al., 2001). Researchers also established that children’s social experience and cognitive development had a profound impact on their emotional comprehension; if children developed better social and cognitive competence than their peers, they can reach higher level of emotional comprehension (Pons et al., 2014). Further, parents’ meta-emotional philosophy and their relationships with children predicted the development of children’s emotional comprehension to a certain extent (Katz and Windecker-Nelson, 2004). Camras and Shuster (2013) showed that emotional comprehension helped to develop social responsiveness, which in turn fostered the ability of communication and emotional resilience; also, children’s emotional comprehension can predict their social adaptation, especially their relationship with peers (Gross, 2002; Ciarrochi et al., 2008; Deneault and Ricard, 2013). It is because children’s understanding of emotions promotes their emotional expression and the formation of thinking. At the same time, it helps children to deal with personal emotions and interpersonal relationships, and reach consensus in peer conflicts (Lu, 2010). Therefore, children with better emotional comprehension have the strength to gain acceptance and build new friendship relationships (Kouvava et al., 2022).

Peer Conflict is usually driven by certain emotional experience in peer interaction, so it is viewed as the result of emotion management (Chen et al., 2001). Researches have pointed out the correlation between peer conflict and emotional comprehension. The main conclusion is that children’s peer conflict plays an important role in development of social competencies, including obtaining an understanding of peers’ feelings and intensions (Licht et al., 2008). However, it requires a more explicit explanation of how preschool children’s emotional comprehension affect peer conflict resolution strategies and the process mechanism. Therefore, the purpose of this study was to investigate the development of emotional comprehension and peer conflict resolution strategies of children aged 3 to 6, and the relationship between them. Based on relevant researches, the following assumptions are made: (a) the effective use of conflict resolution strategies and emotional comprehension of children develop with age, and there are gender differences; (b) emotional comprehension ability is closely related to children’s conflict resolution strategies, and the former can predict the latter.

## 2. Materials and methods

### 2.1. Research design

This study selected preschool children of different ages by random cluster sampling, and evaluated their emotional comprehension and peer conflict resolution strategies. It consists of two parts: First, it examines the gender and age differences of children’s emotional comprehension and peer conflict resolution strategies; Secondly, by controlling children’s gender and age, linear regression method was used to explore the impact of children’s emotional comprehension on peer conflict resolution strategies.

### 2.2. Participants

The original sample consisted of 108 preschool children aged 3 to 6 in Zhejiang Province, and 18 were excluded because they encountered difficulties in completing the tasks. Thus, there were 90 valid participants, 30 in 3-year-old group (*M* = 3.44, *SD* = 0.28, 15 boys), 30 in 4-year-old group (*M* = 4.56, *SD* = 0.32, 15 boys), and 30 in 5-year-old group (*M* = 5.45, *SD* = 0.27, 15 boys). The sample size met the requirements calculated according to G*Power (Erdfelder et al., 1996; Faul et al., 2007, 2009). Before participation, parents of preschool children signed informed consent forms.

### 2.3. Instruments

#### 2.3.1. The test of emotion comprehension

The Test of Emotion Comprehension (TEC) is scored one-to-one by the teachers (Pons et al., 2004, 2014). There are nine components in total, and these components are hierarchically organized into three groups, including external emotional comprehension, mental emotional comprehension, and reflective emotional comprehension. The external emotional comprehension consists of expression recognition, understanding of external causes and of the impact of associated external events or reminders. The mental emotional comprehension includes understanding of beliefs and desires on emotions, and the distinction between outwardly expressed and privately felt emotions. The reflective emotional comprehension focuses on mixed feelings, emotional regulation, and understanding of emotions ensured from morally action (Pons et al., 2004, 2014). According to TEC, the higher the score, the better the child’s emotional comprehension.

#### 2.3.2. The conflict resolution strategy questionnaire

The Conflict Resolution Strategy Questionnaire (CRSQ) was adapted with reference to the research of Liu (2017). There are a total of 10 strategy items, including 5 negative strategies and 5 positive strategies. The items on negative strategies are as follows: “When he/she wants the same toy as another child, he/she would grab it directly,” “He/She often complains to the teacher,” “He/She uses warnings or threats to stop others’ words and deeds,” “When dissatisfied with others in game, he/she would use force to attack companions,” and “When fighting for the right to play, he/she tends to yield.” Meanwhile, the items on the positive strategies include “He/She is able to resolve conflicts through negotiation,” “He/She proposes rock-paper-scissors game to resolve conflicts,” “He/She tends to reason in disagreements,” “He/She can resolve conflicts peacefully,” and “If he/she wants to take toys from others, he/she would propose toy exchange or take turns.” Each item is coded on a 5-point Likert scale including specific frequency ratings (“Never,” “Occasionally,” “Sometimes,” “Often,” “Very often”). The total score of conflict resolution strategies is composed of the sum of the scores for negative and positive strategies, where the negative strategies are scored reversely. Higher scores indicate better conflict resolution strategies in children.

### 2.4. Procedure

TEC was conducted in a quiet, warm and familiar place, such as a book corner or a small balcony in the classroom. According to the principle of random cluster sampling, participants were selected. Before the test started, the researchers conducted a five-day familiarization period with the participants to ensure the smooth progress of the study. When children relaxed, researchers began to test the nine components of TEC. It took 10–15 min for each child. After the test of emotional comprehension, the researchers sent the CRSQ to the teacher of the child participant, and he/she was required to fill the CRSQ based on his/her daily observation and understanding of the child’s behavior.

### 2.5. Statistical analysis

SPSS25.0 was used for statistical analysis, including descriptive statistics, analysis of variance, independent sample t-test, Kruskal-Wallis test, Mann–Whitney U test, correlation analysis and regression analysis.

## 3. Results

### 3.1. Descriptive statistics

The scores of children’s peer conflict resolution strategies and emotional comprehension are shown in Table 1. The former conformed to normal distribution, but the latter non-normal distribution.

**Table 1:** Scores of peer conflict resolution strategies and emotional comprehension in children.

	*M*	*SD*	Skewness	Kurtosis	Kolmogorov–Smirnov
Positive strategies	15.18	3.73	0.54	0.25	0.08
Negative strategies	15.26	2.83	−0.15	−0.47	0.09
Total score of strategies	30.00	5.55	0.27	−0.43	0.07
External	1.88	0.95	−0.32	−0.91	0.20^***^
Mental	0.89	0.87	0.43	−1.02	0.26^***^
Reflective	0.73	0.72	0.82	0.74	0.25^***^
Total score of emotional comprehension	3.50	1.92	−0.02	−0.64	0.15^***^

*** *p* < 0.001.

### 3.2. The developmental characteristics of children’s peer conflict resolution strategies

Taking children’s age and gender as independent variables, and children’s conflict resolution strategies as dependent variables, multiple analysis of variance was performed. The results showed that there was no interaction (all *p*-values > 0.05).

#### 3.2.1. The age differences in children’s peer conflict resolution strategies

The results of one-way ANOVA indicated that there were age differences in children’s strategies selection. Further, the post-hoc analysis suggested that the older children were, the more inclined they were to choose positive strategies (see Table 2). In the use of negative strategies, children in 5-year-old group were significantly lower than those at other ages.

**Table 2:** Results of age difference on children’s peer conflict resolution strategies (M ± SD).

	3-year-old group	4-year-old group	5-year-old group	*F*	*Post-hoc*
Positive strategies	11.73 ± 1.91	15.27 ± 1.96	18.57 ± 3.36	55.92^***^	5 > 4 > 3
Negative strategies	15.43 ± 2.98	16.37 ± 2.74	13.77 ± 2.33	7.17^**^	4,3 > 5
Total score of strategies	26.30 ± 4.02	28.97 ± 4.30	34.83 ± 4.61	30.72^***^	5 > 4 > 3

3 = 3-year-old group; 4 = 4-years-old group; 5 = 5-years-old group.

** *p* < 0.01;

*** *p* < 0.001.

#### 3.2.2. The gender differences in children’s peer conflict resolution strategies

The results of independent sample t-test showed that there were significant gender differences in strategies selection between boys and girls. Girls used more positive strategies, and boys tended to use more negative strategies (see Table 3).

**Table 3:** Results of gender difference on children’s peer conflict resolution strategies.

	Boys	Girls	*t*	*p*
Positive strategies	14.22 ± 3.44	16.16 ± 3.82	−2.52	0.013
Negative strategies	15.78 ± 3.07	14.60 ± 2.57	1.97	0.051
Total score of strategies	28.44 ± 5.53	31.62 ± 5.20	−2.81	0.006

### 3.3. The developmental characteristics of children’s emotional comprehension

#### 3.3.1. The age differences in children’s emotional comprehension

The results of Kruskal-Wallis test (see Table 4) showed that there were age differences in the medians of the total score and each dimension of children’s emotional comprehension, and the scores of children in the 3-year-old group were the lowest.

**Table 4:** Results of age difference on children’s emotional comprehension.

	3-year-old group	4-year-old group	5-year-old group	Kruskal–Wallis	*Post-hoc*
External	1(0.00)	2(0.50)	3(0.50)	44.64^***^	5,4 > 3
Mental	0(0.50)	1(1.00)	1(0.50)	21.74^***^	5,4 > 3
Reflective	0(0.13)	1(0.50)	1(0.13)	24.60^***^	5,4 > 3
Total score of emotional comprehension	1(1.00)	4(1.00)	5(1.00)	48.51^***^	5,4 > 3

Data is presented as median (quartile deviation). ***p < 0.001.

#### 3.3.2. The gender differences in children’s emotional comprehension

The Mann–Whitney U test results (see Table 5) indicated that there was no gender difference in emotional comprehension.

**Table 5:** Results of gender difference on children’s emotional comprehension.

	Boys	Girls	*U*	*p*
External	2(1.00)	2(1.00)	994.50	0.879
Mental	1(1.00)	1(1.00)	989.00	0.840
Reflective	1(0.50)	1(0.50)	986.50	0.817
Total score of emotional comprehension	4(1.50)	4(1.50)	1006.50	0.961

### 3.4. The influence of children’s emotional comprehension on peer conflict resolution strategies

Through correlation analysis (see Table 6), it was found that external emotional comprehension, mental emotional comprehension, and reflective emotional comprehension were positively correlated with positive strategies and the total score of peer conflict resolution strategies, and negatively correlated with children’s negative strategies.

**Table 6:** Correlation analysis results of children’s peer conflict resolution strategies and emotional comprehension.

	Positive strategies	Negative strategies	Total score of strategies
External	0.61^**^	−0.36^**^	0.60^**^
Mental	0.47^**^	−0.51^**^	0.59^**^
Reflective	0.45^**^	−0.28^**^	0.45^**^
Total score of emotional comprehension	0.67^**^	−0.52^**^	0.73^**^

**p < 0.01.

The results of linear analysis (see Table 7) showed that after controlling children’s gender and age, children’s external, mental and reflective emotional comprehension could positively predict the total score of peer conflict resolution strategies and negatively predict their negative resolution strategies. Additionally, the mental emotional comprehension could positively predict children’s positive resolution strategies.

**Table 7:** Regression analysis results of emotional comprehension on children’s peer conflict resolution strategies.

	Positive strategies	Negative strategies	Total score of strategies
Gender	−0.26^***^	0.19^*^	−0.28^***^
3-year-old group or not	−0.78^***^	−0.43^**^	−0.18
4-year-old group or not	−0.39^***^	0.20^*^	−0.32^***^
Mental	0.15^*^	−0.50^***^	0.35^***^
External		−0.35^**^	0.30^**^
Reflective		−0.28^**^	0.23^**^
*R^2^*	0.65	0.53	0.69
∆*R^2^*	0.02	0.35	0.20
*F*	39.12^**^	15.66^**^	31.39^**^

The R^2^ corresponds to the complete regression, and the ∆*R*^2^ corresponds to the increment after excluding the influence of gender and age. *p < 0.05; **p < 0.01; ***p < 0.001.

## 4. Discussion

### 4.1. Characteristics of children’s peer conflict resolution strategies

#### 4.1.1. Age differences in children’s peer conflict resolution strategies

This study found that with the growth of age, children gradually reduce using negative strategies and adopt positive ones in coping with conflict, which is consistent with the previous researches. Children’s conflict-resolving behaviors are related to their age and maturity (Miller and Olson, 2000). In conflict situation, young children were more inclined to choose negative strategies, such as grab and yielding; however, older children preferred positive strategies, such as negotiation and exchange, which is consistent with the conclusions of Ohbuchi and Yamamoto (1990). Children in the 3-year-old group were still in the self-centered developmental stage, so it is difficult for them to solve conflicts with positive strategies, e.g., empathizing with others’ experiences and feelings in peer conflicts. As children’s social cognitive and communication ability developing with age, they gradually realize that positive strategies play an important role in peer interaction, and are more conducive to their interaction, i. e., children use more positive strategies in peer conflict resolution, and new types of positive strategies are constantly emerging.

#### 4.1.2. Gender differences in children’s peer conflict resolution strategies

The results indicated that boys preferred negative strategies to positive ones, while the opposite was true for girls. Relevant studies have illustrated that there were gender differences in aggressive behavior, withdrawal behavior and exclusion. Girls are more likely to adopt prosocial behavior than boys, and boys tend to use more physically aggressive strategies and withdrawal behavior than girls (Miller et al., 1986; Rose and Asher, 1999; Sadri and Rahmatian, 2003; Spivak, 2016). In other words, prosocial behavior significantly predicts children’s conflict resolution strategies. Girls mature socially earlier than boys, which leads to gender differences in conflict resolution strategies. Further, social expectations and culturally shared norms reinforce gender-specific stereotypes (Eagly, 2009). Girls are trained to be more affective and to minimize aggressive display, whereas boys are expected not to show an overabundance of affection (Kostelnik et al., 2011). Williams and Bybee (1994) noted that the gender role prescriptions compel girls to act considerately to peers, otherwise transgressions lead to feelings of guilt.

### 4.2. Characteristics of children’s emotional comprehension

#### 4.2.1. Age differences in children’s emotional comprehension

Children’s emotional comprehension has age differences and develops with age. The 3-year-olds have a certain ability to understand emotions, which is consistent with previous studies. Pons et al. (2014) documented that the more social experience and cognitive development children had, the higher level of emotional comprehension the children could reach, which partly explained the age differences. As age grows, children’s social cognitive competencies increase, leading to improved social emotional competence, as well as emotional comprehension. Besides, the scores of the three dimensions of emotional comprehension in different age groups indicated that the development of each dimension may have a sequence. Previous study has found that infants respond differently to various facial emotional expressions starting at 7 months old (Vanderwert et al., 2015). Compared with the other two dimensions of emotional comprehension, the mixed emotional comprehension developed the slowest (Rocha et al., 2015). It is illustrated that children are limited by maturity, and lack of cognitive ability has an impact on children’s understanding of mixed emotions (Zajdel et al., 2013). Several studies have confirmed that there are age differences in the development of the components of emotional comprehension, and the differences appear to be stable from age three to six (Brown and Dunn, 1996; Pons et al., 2003). Indeed, children’s emotional comprehension is a gradual development process, and early childhood is an important stage of emotional comprehension development.

#### 4.2.2. Gender differences in children’s emotional comprehension

The present study found that there was no significant difference in the development of emotional comprehension between boys and girls. Researchers also insisted that though boys seemed more aggressive than girls, there was no difference in their knowledge of emotions (Cassidy et al., 1992; Arsenio et al., 2000). The finding is in line with the previous researches (Pons et al., 2014). However, some researchers maintained that girls outperformed boys at understanding complex and mixed emotions. The difference may be due to the fact that the surrounding environment makes girls more sensitive to the feelings of their peers and tend to express their felt emotions (Bosacki and Moore, 2004; Kostelnik et al., 2011; Zajdel et al., 2013). However, the social climate and parenting style play a pivotal role in shaping children’s self-awareness of gender, and subsequent gender-typed behavior (Root and Denham, 2010). Therefore, children aged 3 to 6 may display gender differences in social behavior, but there is no significant difference in emotional comprehension. Emotional comprehension is one of the main social skills in early childhood, and only during the long-term socialization process will the differences between children become obvious. Thus, gender differences in the development of emotional comprehension may not be obvious in early childhood.

### 4.3. The influence of children’s emotional comprehension on peer conflict resolution strategies

Through the research, it was found that the components of children’s emotional comprehension were positively correlated with children’s positive strategies and the total score of peer conflict resolution strategies, and negatively correlated with children’s negative strategies. This means the higher children’s emotional comprehension, the more positive strategies they will use in the peer conflict; If not, they will use more negative strategies. According to previous research, emotional comprehension is the key contextual factor that influence one’s social behavior (Coulombe et al., 2019), which suggests that children with better ability of understanding emotions have better prosocial behavior, including succeeding in peer interaction.

The results of regression analysis showed that children’s emotional comprehension could significantly predict children’s conflict resolution strategies. Specially, the mental emotional comprehension had a positive prediction effect on the positive strategies and negative prediction effect on the negative strategies, and the external and reflective emotional comprehension had a negative prediction effect on the negative strategies. It means children with better mental emotional comprehension, which consists of three components, such as desire-based emotional comprehension, belief-based emotional comprehension and understanding of the possibility of hiding an emotion state, are more inclined to take appropriate ways to resolve peer conflicts, instead of aggressive ways (Camras and Shuster, 2013). However, children’s poor development in any of the emotional comprehension dimensions may lead to the adopt the negative peer conflict resolution strategies.

Emotional comprehension is an important part of social cognitive comprehension, and is regarded as a crucial predictor of development of social competences (de Rosnay and Hughes, 2006; Belacchi and Farina, 2010; Coulombe et al., 2019). Different dimensions of emotional comprehension have different impacts on children’s conflict resolution strategies. Educators and caregivers should pay attention to the specific factors of emotional comprehension that influence the conflict resolution strategies, e, g., when children frequently display negative strategies, it may be because of development delays in external, mental and reflective emotional comprehension. Additionally, the dimensions of emotional comprehension are not fragmented but share a common cognitive basis, and the development of emotional comprehension should lay the groundwork for the mutual improvement of the various components. Social climate and educational programs should be tailored to the development of children’s emotional comprehension, e.g., the facilitation program should focus on the basic dimension for the 3-year-old children, such as expression recognition and external emotional comprehension.

## 5. Limitations and future research

Children’s peer conflict resolution strategies are affected by various factors. Studies have shown that peer conflict resolution strategies are related to their family environment, especially parenting styles (Schudlich et al., 2004), opponents’ reaction (Thornberg, 2006) and adults’ intervention (Blunk et al., 2017). The present study does not consider the influence of these factors on the peer conflict resolution strategies of the subjects, which has certain limitations. The interaction between these factors can be further examined in the future. In addition, this study assessed children’s conflict resolution strategies through teachers’ rating, which is an indirect way and may have certain problems. However, under the comparison of multiple rating approaches, this study determined teacher rating is the better choice. Also, more direct measurement approaches need to be developed in future research, which is also the research direction to be deepened in this study.

## 6. Conclusion

This study found that children’s peer conflict resolution strategies had age differences. With the growth of age, children adopted more positive strategies than negative ones, and girls may use more positive strategies. Children’s emotional comprehension also developed gradually with the age. Moreover, children’s conflict resolution strategies were closely related to their emotional comprehension. Children’s mental, external, and reflective emotional comprehension can positively predict the overall conflict resolution strategies and negatively predicted negative strategies, whereas mental emotional comprehension can positively predict positive strategies.

## Data availability statement

The raw data supporting the conclusions of this article will be made available by the authors, without undue reservation.

## Ethics statement

Ethical review and approval was not required for the study on human participants in accordance with the local legislation and institutional requirements. Written informed consent to participate in this study was provided by the participants’ legal guardian/next of kin.

## Author contributions

HW, DX, and YC designed the research and wrote the manuscript. HW and YL collected and analyzed the data. All authors contributed to the article and approved the submitted version.

## Funding

This study is funded by the Humanities and Social Sciences Research Youth Fund of the Ministry of Education of the People’s Republic of China, (Project No.: 20YJC880001; 18YJCZH199).

## Conflict of interest

The authors declare that the research was conducted in the absence of any commercial or financial relationships that could be construed as a potential conflict of interest.

## Publisher’s note

All claims expressed in this article are solely those of the authors and do not necessarily represent those of their affiliated organizations, or those of the publisher, the editors and the reviewers. Any product that may be evaluated in this article, or claim that may be made by its manufacturer, is not guaranteed or endorsed by the publisher.
